# Towards an extension of equivalent system mass for human exploration missions on Mars

**DOI:** 10.1038/s41526-022-00214-7

**Published:** 2022-08-02

**Authors:** Davian Ho, Georgios Makrygiorgos, Avery Hill, Aaron J. Berliner

**Affiliations:** 1 Center for the Utilization of Biological Engineering in Space (CUBES), Berkeley, CA USA; 2 Department of Bioengineering, University of California Berkeley, Berkeley, CA USA; 3 Department of Chemical and Biomolecular Engineering, University of California Berkeley, Berkeley, CA USA

**Keywords:** Aerospace engineering, Biomedical engineering

## Abstract

NASA mission systems proposals are often compared using an equivalent system mass (ESM) framework, wherein all elements of a technology to deliver an effect—its components, operations, and logistics of delivery—are converted to effective masses, which has a known cost scale in space operations. To date, ESM methods and the tools for system comparison largely fail to consider complexities stemming from multiple transit and operations stages, such as would be required to support a crewed mission to Mars, and thus do not account for different mass equivalency factors during each period and the inter-dependencies of the costs across the mission segments. Further, ESM does not account well for the differential reliabilities of the underlying technologies. The uncertainty in the performance of technology should incur an equivalent mass penalty for technology options that might otherwise provide a mass advantage. Here we draw attention to the importance of addressing these limitations and formulate the basis of an extension of ESM that allows for a direct method for analyzing, optimizing, and comparing different mission systems. We outline a preliminary example of applying extended ESM (xESM) through a techno-economic calculation of crop-production technologies as an illustrative case for developing offworld biomanufacturing systems.

## Introduction

Travel to space is limited by the expense of transporting resources beyond Earth’s gravity well^1^. As a result, early metrics of usability for space systems, especially life support^2^, favored mass as the primary decision factor. Following a request to “provide the designers of future missions with mature technologies and hardware designs, as well as extensive performance data justifying confidence that highly reliable Advanced Life Support Systems (ALS) that meet mission constraints can be developed” by the 1997 NASA Research Council (NRC)^3^, the scope of the Equivalent System Mass (ESM) framework was broadened to account for differences in the cost of resources^4^. The general principle behind this early metric was to calculate the mass of all of the resources required to make the system work. ESM was expanded from theory^5^ to the practice of accounting for processes ranging from controls^6^, agriculture^7^, and recycling^8,9^. Currently, ESM remains the standard metric for evaluating ALS technology development^8,10,11^ and systems^12–15^. It has been adopted for use in trade studies^16–18^, as the metric for life support sizing^19–21^, and has been incorporated into several tools^22–24^.

In its current form^25^, the total ESM ${\mathfrak{M}}$ is defined only for the operations at a specific location as the sum over the set of all systems as

1 $${\mathfrak{M}}={L}_{\rm{eq}}\mathop{\sum}\limits_{i=1}^{\mathcal{A}}\left[\underbrace{\left({M}_{i}\cdot {M}_{\rm{eq}}\right)+\left({V}_{i}\cdot {V}_{\rm{eq}}\right)+\left({P}_{i}\cdot {P}_{\rm{eq}}\right)+\left({C}_{i}\cdot {C}_{\rm{eq}}\right)}_{{{\mathfrak{M}}}_{{{{\rm{NCT}}}}}}\;+\; \underbrace{\left({T}_{i}\cdot D\cdot {T}_{eq}\right)}_{{{\mathfrak{M}}}_{{{{\rm{CT}}}}}}\right]$$

for subsystem $i\in {{{\mathcal{A}}}}$ of the ESM excluding crew-time ${{\mathfrak{M}}}_{{{{\rm{NCT}}}}}$ and the ESM including crew-time ${{\mathfrak{M}}}_{{{{\rm{CT}}}}}$ where *M*_*i*_, *V*_*i*_, *P*_*i*_, *C*_*i*_ are the initial mass [kg], volume [m^3^], power requirement [kW_*e*_], and cooling requirement [kg/kW_th_], *D* is the duration of the mission segment [sol], *T*_*i*_ is the crew-time requirement based on an astronaut crew-member (CM) [CM-h/sol], *M*_eq_ is the stowage factor for accounting for additional structural masses for a subsystem such as shelving [kg/kg], *V*_eq_ is the mass equivalency factor for the pressurized volume support infrastructure [kg/m^3^], *P*_eq_ is the mass equivalency factor for the power generation support infrastructure [kg/kW_*e*_], *C*_*eq*_ is the mass equivalency factor for the cooling infrastructure [kg/kW_th_], *T*_eq_ is the mass equivalency factor for the crew-time [kg/CM-h], and *L*_eq_ is the location factor for the mission segment [kg/kg] which accounts for the cost to transport mass from one location in space to another (such as Earth orbit to Martian orbit). Mass equivalency factors (*V*_eq_, *P*_eq_, *C*_eq_, *T*_eq_) are used to convert the non-mass parameters to mass. While the ESM framework^25^ has been widely adopted in Environmental Control and Life Support Systems (ECLSS) analysis^23,26–29^, it has faced critique for the ambiguity in its application as well as its difficulty in accounting for development costs^30^ and uncertainty^31^. Alternative frameworks have been proposed to replace^32^ or extend ESM with additional metrics that factor in complexity^33^. Given the widespread use of ESM, we believe that the framework should be improved with the addition of missing elements rather than replaced completely.

Previous efforts to quantify the cost in problems of mission-planning/space logistics have relied on metrics based solely on the Initial Mass to Low Earth Orbit (IMLEO)^34,35^ for constant commodity supply and demand^36^ or on carry along mass^37^. In such logistics frameworks like SpaceNet^38–40^ and HabNet^41^, the cost is kept simple to allow for the analysis of complex mission architectures with multiple mission segments. Comparatively, ESM has been most fully developed for ECLSS where the costs of capital equipment, power, operations, transport, and other things have been captured on a common unit scale of mass. While it provides a method for summing the weighted terms of many subsystems, there is no explicit ESM equation that captures total mission costs across systems in various stages of a complex mission^30^. Thus the standard ESM approach faces limitations in that there (1) exists no explicit language for capturing the set of all segments and (2) there exists interdependent relationships between the decision variables within separate segments. Here, we see a trade-off in the complexity of the cost function for the complexity of the mission architecture.

As plans for human exploration continue to be made in anticipation of returning to the moon^42^ and traveling to Mars^43,44^, an added emphasis will be required for the optimization of mission architecture^40^. As of now, the current instance of the ESM framework does not lend itself to use as an objective function in optimization over a mission—although this ESM has been proposed as the metric for mission optimization^45^. The result is that this standard framework remains fixed for multi-stage missions and generally (but not always^27^) faces challenges in providing design or planning information based on subsystem risk. Thus, the ESM metric is not always helpful when comparing missions with differential reliability for systems in their proper context. That is, given two possible technologies for meeting a mission objective, the one that is less likely to fail might be a better choice. To demonstrate how to formally add reliability metrics to the ESM framework, we take the case of a new technology platform, biomanufacturing^44,46,47^, for which there are known and quantifiable reliability concerns and for which there are little in situ testing for space missions. In the following work, we propose an extended ESM (xESM) framework to account for the proposed multi-stage missions and critical mission features, such as reliability. As the scope of human exploration missions has expanded, the need for new technology platforms has grown, and it has been proposed that these features best capture the potential of biomanufacturing systems^44^. We do not claim completion of xESM, but rather, we demonstrate progress along this trajectory in the form of a more generalized framework to (1) account for multi-staged mission segments (beyond simple summation); (2) account for reliability; and (3) feed into downstream optimization problems. We also note that this later progress is less developed in more in line with a discussion rather than a ready-to-use operational strategy.

## Perspective

### Extending ESM for long-duration mission profiles

**Fig. 1:**
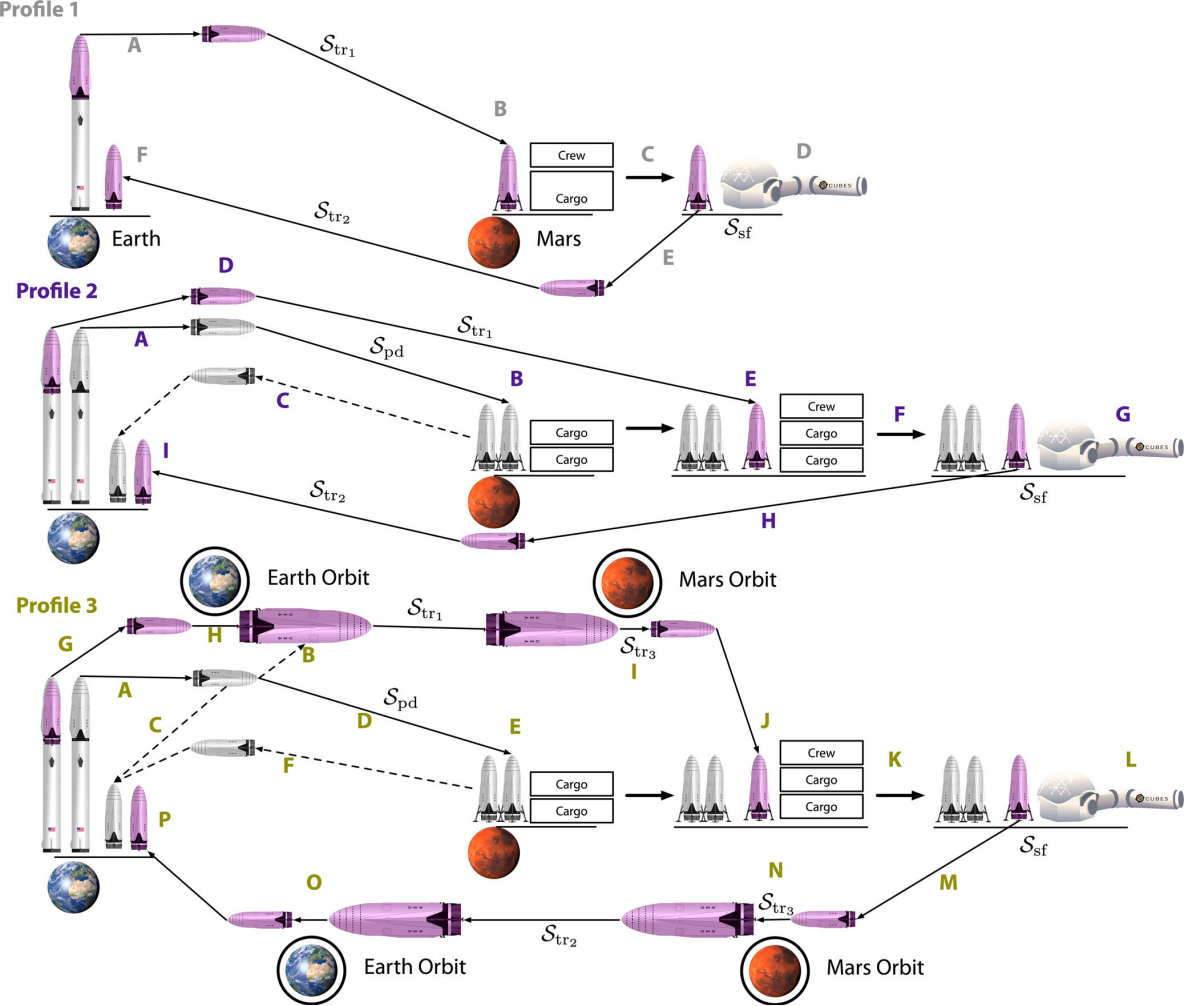
Transit diagram of proposed mission architecture. In Profile 1 (gray), **A** a crewed transit ship is launched directly from the surface of Earth and **B** lands on the surface of Mars where **C** the crew assembles the cargo in habitat and carries out **D** surface operations until **E** the crew launches from their initial transit ship from the surface of Mars into space and **F** lands back on the surface of Earth. In Profile 2 (purple), **A** cargo transit ships without crew are launched directly from the surface of Earth and **B** land on the surface of Mars where cargo can be unloaded. In the case of reusable rocket systems^73^, **C** the cargo rockets can be launched from Mars and returned to Earth. Once all the cargo has been loaded onto the surface of Mars, **D** a crewed transit ship is launched directly from the surface of Earth and **E** lands on the surface of Mars where **F** the crew assembles the cargo in habitat and carries out **G** surface operations until **H** the crew launches from their initial transit ship from the surface of Mars into space and **I** lands back on the surface of Earth. In Profile 3 (green), a number of **A** cargo transit ships without crew are launched directly from the surface of Earth and either **B** supply a previously interplanetary rocket then **C** return to the surface of Earth or **D** travel to the surface of Mars where **E** cargo can be unloaded. In the case of reusable rocket systems, **F** the cargo rockets can be launched from Mars and returned to Earth. Once all the cargo has been loaded on the surface of Mars, **G** a crewed transit ship is launched directly from the surface of Earth to Earth Orbit **H** where it rendezvous with an interplanetary rocket which **I** travels to Martian orbit. The crew **J** then boards a descent vehicle and lands on the surface of Mars where **K** the crew assembles the cargo in habitat and carries out **L** surface operations until **M** the crew launches from their initial transit ship from the surface of Mars into **N** Martian orbit where they again rendezvous with their interplanetary rocket which travels to **O** Earth orbit at which point they board a descent rocket in which they **P** finally return to the surface of Earth.

Figure 1 depicts three profiles with varied transit architectures. Profile 1 (gray) uses a single journey from Earth to Mars, and although it has been proposed in some forms^48^, it is unlikely this architecture will be adopted due to the substantial mass demands of the transit ship and the ascent propellant required to leave Mars^49^. In the case of Profile 2 (purple), cargo can be predeployed to Mars through some number of predeployment missions. Profile 2 introduces segments to a crewed mission to Mars which are not actually crewed, but instead are either purely cargo-based in which case only the *M* and *V* terms factor into the ESM cost, or autonomous where *M*, *V*, *P,* and *C* for uncrewed operations matter. Since cargo missions do not require life support systems, the *M* cost is reduced greatly^12^, leading to a reduction in overall mission cost, especially for missions that require a great number of goods that can be predeployed. In the most likely Profile 3^50,51^ (green), crew transportation can be further broken down such that smaller crewed vehicles make the jump from planet to surface and vice-versa, but the interplanetary transit is made on a larger craft to reduce the mass required for egress from planetary gravity wells.

Previous ESM literature allows for varied equivalency factors based on mission staging^25^, and in such cases, the ESM of distinct segments of a mission are calculated separately, then normalized through the use of location factors^52^. However, ESM ${\mathfrak{M}}$ for any set of systems is calculated using a single location factor *L*_eq_ term as a multiplier. In this form, it is assumed that each subsystem is transported in a uniform fashion or that all parts of a subsystem would correspond to a single *L*_eq_ term. The profile expansion in Fig. 1 shows that inventory can be transported in different segments using different crafts which changes the value of *L*_eq_. This is supported by non-ESM logistics methods^40^. We argue that the use of predeployment missions for transporting cargo implies that a system on one particular segment may utilize components transported from multiple segments, each with different location factors, motivating a more generalized articulation of xESM (${{\mathfrak{M}}}_{0}$) as

2 $${{\mathfrak{M}}}_{0}=\mathop{\sum }\limits_{k}^{{{{\mathcal{M}}}}}\underbrace{L_{\mathrm{eq},k}\sum_{i}^{{\mathcal{A}}_{k}}{\left[({M}_{{k}_{i}}\cdot {M}_{{{{\rm{eq}}}},k})+\left({V}_{{k}_{i}}\cdot {V}_{{{{\rm{eq}}}},k}\right)+\left({P}_{{k}_{i}}\cdot {P}_{{{{\rm{eq}}}},k}\right)+\left({C}_{{k}_{i}}\cdot {C}_{{{{\rm{eq}}}},k}\right)+\left({T}_{i}\cdot {D}_{k}\cdot {T}_{{{{\rm{eq}}}},k}\right)\right]}}_{\begin{array}{c}{{\mathfrak{M}}}_{0,k}\end{array}}$$

3 $$={{\mathfrak{M}}}_{0,{{{\rm{pd}}}}}+{{\mathfrak{M}}}_{0,{{{\rm{sf}}}}}+{{\mathfrak{M}}}_{0,{{{{\rm{tr}}}}}_{1}}+{{\mathfrak{M}}}_{0,{{{{\rm{tr}}}}}_{2}}+{{\mathfrak{M}}}_{0,{{{{\rm{tr}}}}}_{3}}$$

where ${{{\mathcal{M}}}}$ is sum of ESM for segments in a mission set with index *k*. Mission segment ${{{\mathcal{S}}}}$ can be constructed via set-builder notation as ${{{\mathcal{S}}}}=\{(i,j)| i\in {{{{\mathcal{L}}}}}_{2};j\in {{{\mathcal{O}}}}\}$ for specific combinations of locations and operations (see Methods for additional definitions). Essentially, we have established a graph where the locations represent nodes and the segments represent arcs, which matches previous formulations of mission logistics^40^, although our set of location nodes is reduced for simplicity and does not include specific Lagrange Points^34^. The generalization enables the accounting of mission segment-specific terms such as location factor *L*_eq_ and equivalency factors (*M*_eq_, *V*_eq_, *P*_eq_, *C*_eq_, *T*_eq_). This generalization also allows for indexing of mission segment-specific subsystems ${{{\mathcal{A}}}}$, further enabling an accounting of inventory ${{{\mathcal{I}}}}$ elements between mission segments ${{{\mathcal{S}}}}$.

Since these developments have been primarily applied to longer-duration ECLSS systems for the International Space Station (ISS) and not Mars missions, xESM does not include recent developments in resupply logistics^53^ as enabled by the decreasing cost to LEO^54^. Despite a decreased cost to LEO, resupply logistics will be unlikely to impact the initial set of crewed exploration missions^49^ given the difference in resupply costs between the  and  systems. Although arguments have been raised against the adoption of crew-time within the ESM^55^, we include these terms in our formulation as it has been the standard.

#### Inventories and dependent factors

**Fig. 2:**
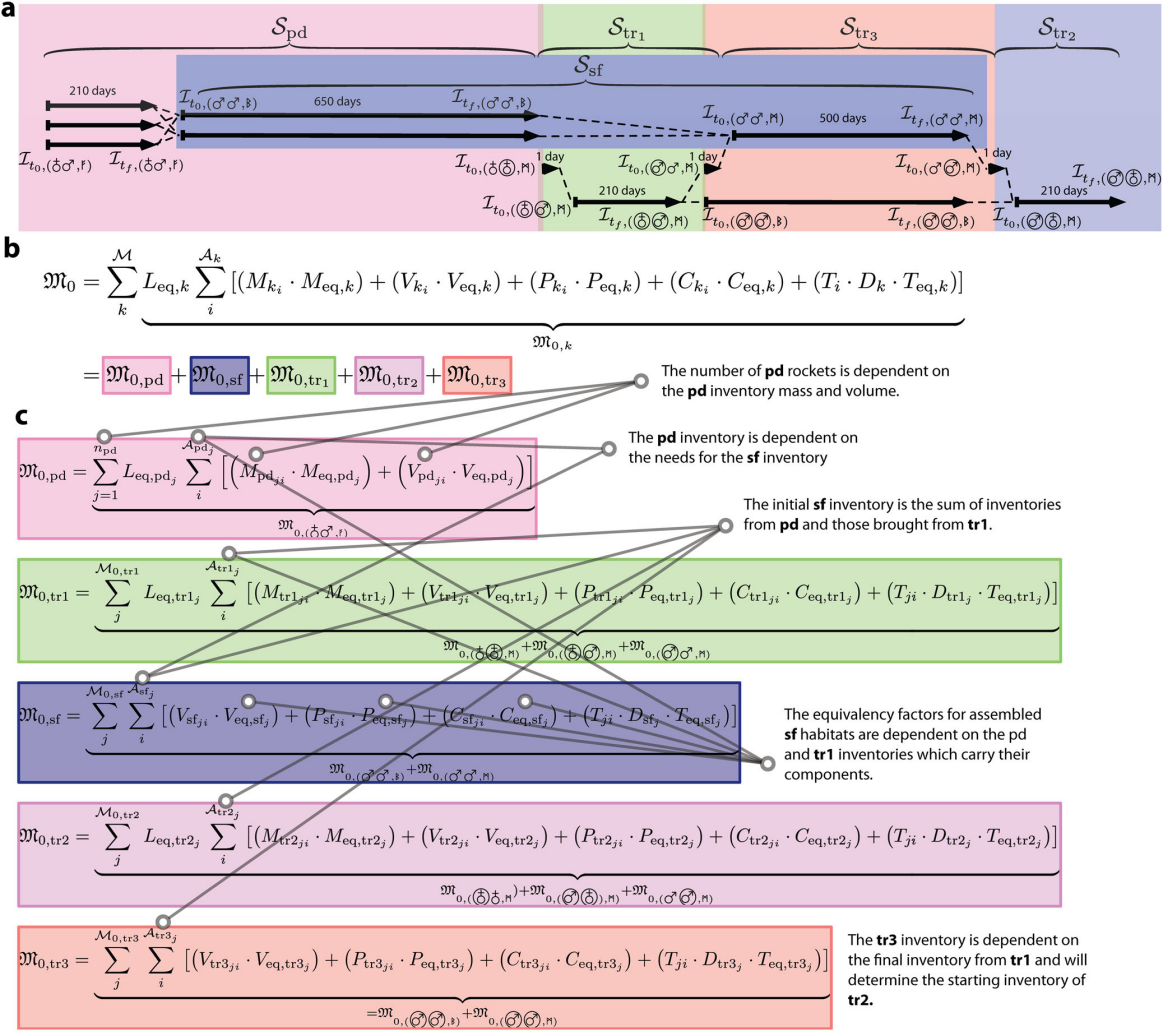
xESM equation for Profile 3 (Fig. 1) with terms decomposed by subsystem. **a** Breakdown of inventory transfers across mission timeline colored by mission segment. **b** The generalized xESM equation colored by mission segment. **c** Expanded xESM equation with colored by mission segment with a non-exhaustive set of specific segment-dependent relationships elucidated. Legend: Predeployment (pd): pink. Legend: Transit 1 (tr_1_): yellow. Transit 2 (tr_2_): blue. Transit 3 (tr_3_): orange. Surface operation (sf): purple.

With the addition of our method for indexing factors by their location, operation, and hardware, we are now able to address the accountancy of relationships between equivalency/location factors and the segment inventory that defines them. In essence, equivalency/location factors convert non-mass properties to mass properties by means of a ratio, but because that mass originates from some subset of inventory elements, equivalency and location factors are coupled. The exact nature of this interaction depends on the scenario and the modeling itself, and we aim to present a preliminary rendering of these relationships in Fig. 2. In our assumptions, we say that predeployment cargo is grouped into cargo shipments in set *j* of ${{{{\mathcal{M}}}}}_{{{{{\rm{pd}}}}}_{j}}$ across some number of predeployments *n*_*pd*_. We assume that this set of cargo is composed of items such as habitat assemblies, control hardware, photovoltaics & batteries, reactors, tanks refrigerators, various experimental apparatus, 3D printers, and other tools^12^. In the more expanded surface operations term, Fig. 2 demonstrates that the inventory for surface operations is composed of an assembled habitat, process and reactor assemblies, mission crew, and integrated power systems. In this scenario, a set of equivalency factors are required for each segment of the mission.

The location factor *L*_eq_ is the reciprocal of the payload fraction for transporting mass between two points in space and can be evaluated as the sum of across multiple orbital maneuvers with different Δ*v*. Each element in the location mapping ${{{{\mathcal{L}}}}}_{2}$ has a specific required Δ*v*. Any segment describing operations in a single location, such as Martian surface operations, has no mass transport and thus will have a *L*_eq_ = 1.0. Since Δ*v* can be related to the specific impulse *I*_sp_ and mass fraction *m*_0_/*m*_*f*_ via the Tsiolkovsky rocket equation^1^, we see how the mass of a specific segment inventory affects the location factor term. In terms of specific calculations, the mass fraction is the ratio of the of initial total rocket mass *m*_0_ to final total mass *m*_*f*_, and the payload fraction is the ratio of initial total mass *m*_0_ to final delivered mass *m*_*p*_ (no propellant, tanks, etc). Meanwhile, the *m*_0_, *m*_*f*_, and *m*_*p*_ will be constrained by rocket technology choice. The scaling of the location factor is nonlinear in the case where some number of predeployments are each limited in payload mass. We calculate the ${{\mathfrak{M}}}_{0,{{{\rm{pd}}}}}$ as the sum over the number of total predeployments *n*_pd_ where a given predeployment *j* has a set of cargo ${{{{\mathcal{I}}}}}_{{{{{\rm{pd}}}}}_{j}}$ that doesn’t require *V*, *P*, or *T*. The number of predeployment rockets will be parametric based on the *m*_*p*_ for predeployment rockets and the sum of all inventory mass to be used on the martian surface shipped by predeployment. As shown in Fig. 2, the ${L}_{{{{{\rm{eq,pd}}}}}_{j}}$ in the ${{\mathfrak{M}}}_{0,{{{\rm{pd}}}}}$ term can be related to the *M* and *V* terms for the components of predeployment *j*, while the ${L}_{{{{{\rm{eq,tr}}}}}_{1}}$ and ${L}_{{{{{\rm{eq,tr}}}}}_{2}}$ terms are related to the *M* and *V* for all cargo transported in the complete mission.

Like *L*_eq_, equivalency factors are also parametric based on certain elements of a segment inventory as showed by the cross-dependent mission-segment network (Fig. 2c). For example, the volume equivalency *V*_eq_ for crewed transits in space will be based on the pressurized volume^52,56^ of the vehicle. Our notation affords the specification of equivalencies with relation to other decision variables, as opposed to the cruder method of assigning general constants. Figure 2 illustrates how the equivalency factors for one segment will often be parametrically related to decision variables in other segments. This realization only enforces the importance of our extension by which multiple segments are represented by a single optimization metric.

#### Example calculations

**Fig. 3:**
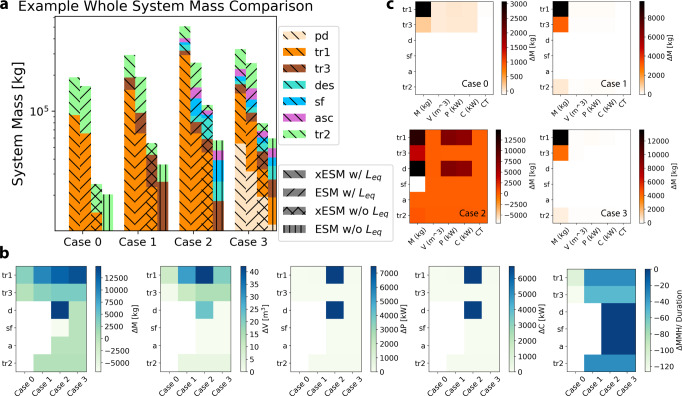
Comparison of ESM and xESM metrics for whole system mass scenarios. **a** Log-scale comparison of mission segment mass for increasing mission assembly. Case 0 is a baseline inventory for the flight from Earth orbit to Mars orbit (tr_1_) and back (tr_2_), while life support for a 500 day Mars orbit (tr_3_) is added in Case 1. The mission in Case 2 includes descent (des), Mars surface operations (sf), and ascent (asc). All inventory for sf is predeployed (pd) in Case 3. As the mission grows, both the mass required and the difference between xESM and ESM increases. The final case shows falling xESM with the removal of sf inventory from tr_1_ and des. **b** Inventory difference between xESM and ESM in raw mass, volume, power, cooling, and crew-time across each mission segment, before the application of location and equivalency factors. **c** Raw inventory difference between xESM and ESM displayed across the four cases. Legend: Predeployment (pd): tan. Transit 1 (tr_1_): orange. Transit 2 (tr_2_): yellow. Transit 3 (tr_3_): red. Surface operation (sf): blue. Descent (des): gray. Ascent (asc): purple. Extended ESM with Location Factor (xESM w/L_eq_): back-slash. Standard ESM with Location Factor (ESM w/L_eq_): forward-slash. Extended ESM without Location Factor (xESM w/o L_eq_): cross slash. Standard ESM with Location Factor (ESM w/o L_eq_): vertical line.

**Fig. 4:**
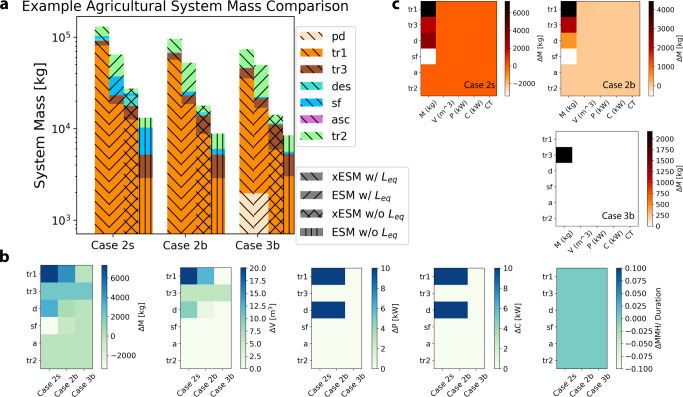
Comparison of ESM and xESM metrics focused on the stored food cost. **a** Log-scale comparison of mission segment mass for different food strategies. Case 2s is the food cost of Case 2 in Fig. 3. Case 2b reduces the amount of stored food for sf from 500 days to 70 days, assuming a hypothetical future agriculture system could grow the difference. In Case 3b, the sf inventory is predeployed, and grown food also sustains the majority of sf. The ESM differences between 2s and 2b and between 2s and 3b show the rough mass requirement for the design and development of such an agricultural system. **b** Raw inventory difference between xESM and ESM mass, volume, power, cooling, and crew time across each mission segment, before the application of location and equivalency factors. **c** Raw inventory difference between xESM and ESM displayed across the three cases. Legend: Predeployment (pd): tan. Transit 1 (tr_1_): orange. Transit 2 (tr_2_): yellow. Transit 3 (tr_3_): red. Surface operation (sf): blue. Descent (des): grey. Ascent (asc): purple. Extended ESM with Location Factor (xESM w/L_eq_): back-slash. Standard ESM with Location Factor (ESM w/L_eq_): forward-slash. Extended ESM without Location Factor (xESM w/o L_eq_): cross slash. Standard ESM with Location Factor (ESM w/o L_eq_): vertical line.

To illustrate the process for calculating xESM with both the traditional approach and our proposed method, we provide the following example problems. The first explores a calculation across all inventory systems of a mission (Fig. 3) and the second has been scoped to the food production (Fig. 4) using Controlled Ecological Life Support Systems (CELSS)^57,58^, which we feel serves as an established and graspable biomanufacturing-based technology^44^ for comparison against “bring everything” or physical/chemical life support systems^59^.

The first example is offered to demonstrate a broad comparison between ESM/xESM, and in Case 0, we represent a base mission with the corresponding inventory required to fly from Earth orbit to Mars orbit (${{{{\mathcal{S}}}}}_{{{{{\rm{tr}}}}}_{1}}$: 210d, 6CM) and back (${{{{\mathcal{S}}}}}_{{{{{\rm{tr}}}}}_{2}}$: 210d, 6CM), and we assume the orbital mechanics allow for this transit. In Case 0, the inventory elements in a craft are scaled for their entire duration of use (420d), and consumables (food, waste collection, water) are used or discarded as time passes. In Case 1, we build on the base case by including the segment in which the crew would orbit Mars (${{{{\mathcal{S}}}}}_{{{{{\rm{tr}}}}}_{3}}$: 210d, 6CM); and like in the previous case, items in a craft are scaled for their entire duration of use (920d). In Case 2, we continue to build on the previous case by including descent (${{{{\mathcal{S}}}}}_{{{{\rm{dec}}}}}$: 500d, 4CM), surface operations (${{{{\mathcal{S}}}}}_{{{{\rm{sf}}}}}$: 500d, 4CM), and ascent (${{{{\mathcal{S}}}}}_{{{{\rm{asc}}}}}$: 1d, 4CM). Here the *M*, *V*, *P*, *C* inventory terms needed for ${{{{\mathcal{S}}}}}_{{{{\rm{sf}}}}}$ are carried in ${{{{\mathcal{S}}}}}_{{{{{\rm{tr}}}}}_{1}}$ and ${{{{\mathcal{S}}}}}_{{{{\rm{dec}}}}}$ (with crew-time requirements for these items not accounted for). Here, 4 crew-members are left in orbit on ${{{{\mathcal{S}}}}}_{{{{{\rm{tr}}}}}_{3}}$. In calculating xESM, the *M* term for ${{{{\mathcal{I}}}}}_{{{{\rm{sf}}}}}$ is ignored during ${{{{\mathcal{S}}}}}_{{{{\rm{sf}}}}}$, as no mass is “moved” during this segment as it was previously transported to the surface via ${{{{\mathcal{S}}}}}_{{{{{\rm{tr}}}}}_{2}}$ and ${{{{\mathcal{S}}}}}_{{{{\rm{dec}}}}}$; additionally, ${{{{\mathcal{S}}}}}_{{{{\rm{asc}}}}}$ is assumed only to transport crew-members back to orbit. In Case 3, we achieve the proposed Profile 3 architecture from Fig. 1 where the surface mission inventory is supplied via predeployment (${{{{\mathcal{S}}}}}_{{{{\rm{pd}}}}}$) rather than the initial transit and decent. Calculations of system mass (ESM and xESM) in Fig. 3 show the expected increase in cost moving from Case 0 to Case 2 in which the size of the inventory grows in relation to the complexity of the situation (see SI for details). Also as expected, and without factoring in the location factor, the use of predeployments in Case 3 reduces the xESM cost by ~35% while only reducing ESM cost by ~1.1% (Fig. 3a). Factoring in location factor, the use of predeployments in Case 3 reduces the xESM cost by ~29% while also increasing the cost by ~3.8% (these percentages are found using the formula for percent change, [final−initial]/[initial], see SI for more details). As the mission scope grows, both the mass required and the difference between xESM and ESM increases as outlined by Fig. 3b, c.

The three Cases in Fig. 4 consider the food system and the potential impact of agricultural biotechnology to supply astronauts with their caloric and nutritional needs. We assume that each of six CMs has a daily dry mass food requirement of 0.617kg/CM-d^12^. We use this requirement to calculate the prepackaged food requirements of the two transit legs of each mission scenario, as well as the extra 70 or 500 days of food for surface operations in Cases 2s and 2b respectively. Given the recently updated infrastructure costs^12^ associated with a Mars Surface Habitat Vehicle^19^, we calculate ESM through consideration of the food subsystem including food, packaging, refrigeration^12,19^, and processing. In Case 2s, we consider only the stored food requirements from Case 2 from Fig. 3. In Case 2b, we consider the stored food requirements during surface operations decreased from 500d to 70d and the remaining food was produced via agriculture. In a long-duration mission scenario in which food is grown during surface operations, and where literature suggests that a sizable initial hardware set would be required^12^. This set could include hydroponic growth chambers, water filtration, refrigeration, etc. along with additional support hardware like pumps, filters, etc^12^. In Case 3, we consider the transportation of the biomanufacturing system during predeployment rather than with the crew. During initial transit as well as the return transit, the crew relies on prepackaged food—crop growth begins on the first day of surface operations, necessitating another ~70 days of predeployed food while the surface hardware grows the first crop^12^. Variations in crop selection and growth conditions during surface operations have been proposed, but this bounding assumption is consistent with crops such as lettuce and wheat^12,57,60^.

Like Cases 0–3, xESM costs for Cases 2s, 2b, and 3b are larger than their ESM alternative, however, in Case 2s (w/o biomanufacturing, only ‘bring everything’) and Case 2b (w/ biomanufacturing), the xESM option is significantly larger than the ESM option for calculation. The difference between the xESM and ESM calculation results is an increased mass on the transit to Mars and reduced mass for surface operations and return transit. The primary trade-off here is that xESM provides a higher fidelity model for multi-segmented missions given that it includes the costs for all mission segments where an item is carried, while the ALSSAT’s ESM calculation method does not include preceding mission segments ALSSAT^19^. This result is especially important considering downstream biomanufacturing options which show a reduced xESM metric in scenarios where predeployment is leveraged to reduce the cost associated with the transit. Additionally, our “bring everything” mission which does not rely on biomanufacturing yields larger costs overall from increased stored food. All three scenarios have equivalent tr_2_ ESM and xESM; this shows that in the last leg of the journey, or in a segment that is not influenced by future operations, ESM equals xESM. While simplified, this captures many of the critical features necessary to demonstrate the need for ESM extension. In cases where inventory from one segment can be used to satisfy constraints in another segment, the ESM summation of separately optimized mission segments can be less optimal than an ESM optimized with an objective function that accounts for both segments and constraint functions containing both terms from both segments. Given that system mass analyses are often used in the preliminary evaluation of technologies, it becomes more important when considering biomanufacturing platforms to leverage the xESM formulation to provide higher fidelity and more favorable metric. However, we also must clarify that the aim of exploring this example is not to make claims about a specific technology, but rather to provide an example for differentiating ESM and xESM.

### Towards xESM analysis and optimization under uncertainty

So far, we have looked at the xESM framework for calculating segmented costs. Based on the scenario chosen, the xESM metric is ultimately determined based on some set of specific technologies that are used. Simpler cases, as the ones given in the examples assume that (1) the behavior of a particular system is fully known on Mars and (2) the operation of the systems is undisturbed by external factors. Although several systems can reliably be considered deterministic in this scope, effects such as micro-gravity might affect the dynamics of specific processes in a biomanufacturing context. Moreover, each process possesses a set of faulty states, i.e., technical issues may cause a system to underperform significantly. Detailed analysis of novel systems, e.g., in the biomanufacturing case, requires the description of the operation of systems using mathematical models. To this end, the xESM framework can be used both to analyze the cost of individual processes as well as the cost of integrated processes in any desired segment, as they operate in time. A simulation-based analysis, either some cost analysis of specific elements or some end-to-end optimization procedure, makes use of models to simulate the systems, the environment, and associated costs for achieving the mission objectives. As a remark, we should note that the sophistication of the simulated case study can vary. For instance, higher-level decisions can be optimized without the need for detailed models for individual components, while exact scheduling^61^ and operational decision-making should involve dynamical models for the various subsystems^62^. This principle has been widely adopted in manufacturing settings for design and control. Parts of the costs not commonly accounted for in cost calculations for space missions like ESM are uncertainty and risk. The latter are important factors during the design phase as we need to ensure safety in a robust, worst-case setting^63^.

Uncertainty can be broken down categorically into two groups: aleatory^64^ and epistemic^65^. Aleatory uncertainties are random and stochastic in nature and, although they can be examined via systematic testing, they cannot be reduced below some threshold. On the other hand, epistemic uncertainties can be reduced by applying additional knowledge and testing much more effectively. Moreover, uncertainties can be categorized and modeled as time-varying and time-invariant. In our case, there are several components, both explicitly and implicitly appearing in the xESM framework, that can be considered uncertain. Let $\theta \in {{\Theta }}\subset {{\mathbb{R}}}_{\theta }^{n}$ denote a vector of uncertainties (both time-varying and invariant). Epistemic uncertainties include time-varying variables such as unmodeled dynamics (e.g., states of the system not taken into account) or time-invariant variables, for example, physical parameters of systems (e.g., kinetic parameters) or operational factors (e.g., the efficiency of lights). Aleatory uncertainties can include purely stochastic dynamics of systems and are typically time-varying while including operational uncertainties related to equipment switching to a faulty state. In our context, note that the multi-segment approach allows for considering segment-specific uncertainties, for example, *θ*_*pd*_ ⊂ Θ are the predeployment-specific uncertainties and *θ*_*sf*_ ⊂ Θ are the uncertainties directly related to the surface operations.

Before formally defining an optimization problem, we should mention that the cost is generally a function of decision variables that reflect design choices regarding the specific utilization of available technology. Let us now focus on a particular segment, i.e., the surface operations and let ${u}_{sf}\in {{\mathbb{R}}}^{n}$ denote a set of decision variables for the surface operations. (e.g., the amount of crop biomass that should be grown over some production cycle or the allocated area for plant growth). The mass-equivalent cost for the surface operations in this case is a function in the form ${{\mathfrak{M}}}_{0,sf}({u}_{sf};{\theta }_{sf})$. The decision variables can be fixed a priori or, more realistically, should be determined upon the solution of an optimization problem that seeks to minimize ${{\mathfrak{M}}}_{0,sf}$ in while accounting for uncertainties. The latter implies that typically we are interested in some expected value of the cost, i.e., ${{\mathbb{E}}}_{{{\Theta }}}\left[{{\mathfrak{M}}}_{0,sf}({u}_{sf};{\theta }_{sf})\right]$. In a more general sense, each segment *j* induces an expected cost ${{\mathbb{E}}}_{{{\Theta }}}\left[{{\mathfrak{M}}}_{0,j}({u}_{j},{\theta }_{j})\right]$. Thus, reliability and uncertainty metrics also should be considered in an optimization setting^66^.

As the entire mission is broken down into segments and sub-segments, we can define task-specific performance level requirements which, when not fulfilled at several points in time, the mission can be considered to be failing. In other words, when simulating some part of the mission, uncertainty can lead to a sequence of faults manifesting themselves (either due to uncertainty in the system dynamics or due to external disturbances and equipment faults) until the mission has to be abandoned. This is a useful definition for incorporating risk into the mission design given the dynamic nature of operations and the breakdown of mission stages that was introduced earlier. Using the notion of segments, we can define as *π*_*t*,*j*_(***θ***_***j***_; *u*_*j*_) the probability density function of segment *j* failing the earliest at time *t*, under some decision variable vector *u*_*j*_. Subsequently, we can rely on sample-based methods to calculate the aforementioned probability, e.g., Monte Carlo sampling. Subsequently, we can define the expected failure time of segment *j* under the set of decisions *u*_*j*_ as ${\hat{t}}_{f}({u}_{j})$ = ${{\mathbb{E}}}_{{{\Theta }}}\left[{\pi }_{t,j}({{{{\boldsymbol{\theta }}}}}_{{{{\boldsymbol{j}}}}};{u}_{j})\right]$, which also reflects the reliability of the design *u*_*j*_. Note that faults and failure are connected but not identical^67^. We define as faults the sequence of events that need to occur such that their accumulation over time (in terms of number and magnitude) lead to an overall failure condition. Therefore, all uncertainties can be propagated into a single indicator which is the time of mission failure, which can be used for further analysis.

We can now shift our attention towards a stochastic optimal decision-making for *u*_*j*_, discussing the elements that would construct a proper stochastic optimization problem^68,69^. The main element is the objective function. In a naive approach, we would seek the design *u*_*j*_ such that the expected segment cost is minimized. Nevertheless, this is not the best approach because we need to account for the confidence in the value of the expected cost. Therefore, the objective should include the variance of the segment cost due to uncertainty, i.e., ${\mathbb{V}}\left[{{\mathfrak{M}}}_{0,j}({u}_{j},{\theta }_{j})\right]$. Last, but not least, a design that causes the segment to fail at a particular day should be incur a penalty to the objective, related to the probability of failure as opposed to the probability of a loss of crew (Pr(LOC))^70^. We can define a scale of that penalty as *s*(*u*_*j*_), which can assume many forms, with the requirement that a mission that lasts longer is penalized less.

Under the simple assumptions that (1) the goal of human exploration missions is to carry out science experiments^49^ and that (2) experiments are carried out each day, a worst-case scenario is a complete mission scrub in which all science objectives are planned beyond the day of mission failure cannot be completed. Overall, the main idea is that if the mission is to fail on the very first days, then it would need to be redone on the following mission. The assumption being made by this simple penalty is that if a mission were to fail early, the ESM cost of that mission left incomplete would be partially added to next one. We argue that this is a valid initial construction of a penalty term based on assumption that incomplete work during a mission is required. This statement is especially valid for early human exploration missions where experimental use of new equipment is important in validating its use or raising technology readiness levels to acceptable values for future missions. While we recognize that the standard recommendation in Decision Theory is to ignore sunk costs, we argue that in our paradigm, this added penalty is not such a sunk cost. In classical decision analysis, a sunk cost is a sum paid in the past that is no longer relevant to decisions in the future^71^ and thus should be ignored when making decisions. We argue that in our paradigm, we are analyzing the impact on a mission of some choice in technology that has some defined uncertainty, and thus no cost has been sunk. In the parlance of decision analysis, this is an example of a prospective cost, and is not to be ignored.

The objective for an optimization problem on a segment can now be written as

4 $$f({u}_{j})={\mathbb{E}}\left[{{\mathfrak{M}}}_{0,j}({u}_{j},{\theta }_{j})\right]+{w}_{v}{\mathbb{V}}\left[{{\mathfrak{M}}}_{0,j}({u}_{j},{\theta }_{j})\right]+{w}_{p}s({u}_{j})$$

where *w*_*v*_ is a weight that assesses the importance of variance of the cost in the objective and *w*_*p*_ is a cost, in system mass units, which, as discussed, attains values approximately equal to a nominal ESM cost for the segment. Moreover, depending on the nature of the problem, the optimization is complemented with various robust constraints. The latter ensures the safe operation of the systems, such as achieving several thresholds of productivity. A detailed optimal decision-making problem formulation is heavily case-dependent and a complex issue to address, however, we envision that the objective function would generally attain this particular in most cases. Last, but not least, the optimization can be extended to a mission-wide horizon by replacing the segment-specific cost with the total cost.

## Outlook

The use of the xESM framework helps guide the development and implementation of software for a reference mission architecture for long-duration human exploration of Mars. We recognize that this extension of ESM as a metric for mission scenario comparison is preliminary and not exhaustive in its scope. We also note that no single analytical result such as ESM or xESM will be the sole factor in the technical specification or platform decision-making. The differences presented are important but modest and are in scale with the uncertainty of the quantities used as the inputs. In addition to the incorporation of mission parameters, specific constants and terms in our formulation are required, such as a more precise calculation of equivalency factors for cooling, power, volume, and crew time and distillation of the specifics for risk fractions. Future endeavors include a comprehensive optimization problem formulation and solution based on the xESM framework both for biologically and non-biologically driven missions. Moving forward, we hope that our extension of ESM provides the basis for continued systems engineering and analysis research for a more quantitative and inclusive design and optimization of long-term human exploration missions.

## Methods

### Mathematics

Let ${{{\mathcal{L}}}}$ be a set of locations composed by  where  is Earth surface,  is low Earth orbit,  is Martian Surface, and  is low Martian orbit. Let ${{{{\mathcal{L}}}}}_{2}$ be the set of pairs in ${{{\mathcal{L}}}}$ which describe from starting to ending location. Let ${{{\mathcal{O}}}}$ be the set of operations composed by  where (Elder Furthark^72^ rune  *fehu meaning “cattle”, used here to imply “cargo”) is cargo, (Elder Furthark rune  *berkanan meaning “tree”, used here to imply “autonomy”) is robotic, and (Elder Furthark rune  *mannaz meaning “man”, used here to imply “crewed”) is crewed. Let Λ(*i*, *j*) be the mapping from some pair of $i\in {{{{\mathcal{L}}}}}_{2}$, $j\in {{{\mathcal{O}}}}$ to the set ${{{\mathcal{R}}}}$ of rockets, vehicles, and habitats. A mission segment ${{{\mathcal{S}}}}$ can be constructed via set-builder notation as ${{{\mathcal{S}}}}=\{(i,j)| i\in {{{{\mathcal{L}}}}}_{2};\,j\in {{{\mathcal{O}}}}\}$ for specific combinations of locations and operations as

5 $${{{\mathcal{S}}}}=\{(i,j)| i\in {{{{\mathcal{L}}}}}_{2};j\in {{{\mathcal{O}}}}\}$$

6789101112131415for the abstract segments of predeployment (pd), crewed transit from Earth to Mars (tr_1_), Martian surface operations (sf), crewed transit back from Mars to Earth (tr_2_), and either autonomous or crewed operations aboard the interplanetary vehicle in Martian orbit (tr_3_). The complete mission object ${{{\mathcal{M}}}}$ is therefore constructed as the collection of these abstract segments in conjunction with the selection of a specific technology in ${{{\mathcal{R}}}}$ as

16 $${{{\mathcal{M}}}}=\{(k,\ell )| k=(i,j)\forall \{{{{{\mathcal{S}}}}}_{{{{\rm{pd}}}}},{{{{\mathcal{S}}}}}_{{{{\rm{sf}}}}},{{{{\mathcal{S}}}}}_{{{{{\rm{tr}}}}}_{1}},{{{{\mathcal{S}}}}}_{{{{{\rm{tr}}}}}_{2}},{{{{\mathcal{S}}}}}_{{{{{\rm{tr}}}}}_{3}}\};\ell ={{\Lambda }}(i,j)\}$$

and can be used in the construction of a generalized total mission ESM ${{\mathfrak{M}}}_{0}$ as

17 $${{\mathfrak{M}}}_{0}=\mathop{\sum }\limits_{k}^{{{{\mathcal{M}}}}}\underbrace{L_{\mathrm{eq}, k}\sum_{i}^{{\mathcal{A}}_{k}}{\left[({M}_{{k}_{i}}\cdot {M}_{{{{\rm{eq}}}},k})+\left({V}_{{k}_{i}}\cdot {V}_{{{{\rm{eq}}}},k}\right)+\left({P}_{{k}_{i}}\cdot {P}_{{{{\rm{eq}}}},k}\right)+\left({C}_{{k}_{i}}\cdot {C}_{{{{\rm{eq}}}},k}\right)+\left({T}_{i}\cdot {D}_{k}\cdot {T}_{{{{\rm{eq}}}},k}\right)\right]}}_{\begin{array}{c}{{\mathfrak{M}}}_{0,k}\end{array}}$$

18 $$={{\mathfrak{M}}}_{0,{{{\rm{pd}}}}}+{{\mathfrak{M}}}_{0,{{{\rm{sf}}}}}+{{\mathfrak{M}}}_{0,{{{{\rm{tr}}}}}_{1}}+{{\mathfrak{M}}}_{0,{{{{\rm{tr}}}}}_{2}}+{{\mathfrak{M}}}_{0,{{{{\rm{tr}}}}}_{3}}$$

as the sum of ESM for segments in a mission set ${{{\mathcal{M}}}}$. Essentially, we have established a graph where the locations represent nodes and the segments represent arcs, which matches previous formulations of mission logistics^40^, although our set of location nodes is reduced for simplicity and does not include specific Lagrange Points^34^. The generalization enables accounting of mission segment-specific terms such as location factor *L*_eq_ and equivalency factors (*M*_eq_, *V*_eq_, *P*_eq_, *C*_eq_, *T*_eq_). This generalization also allows for indexing of mission segment-specific subsystems ${{{\mathcal{A}}}}$, further enabling an accounting of inventory elements between mission segments.

### Example problem calculations

Inventories for the whole system mass in Fig. 3 and the agricultural system mass in Fig. 4 are rendered from ALSSAT^19^ calculation outputs for a Closed Loop (Air and Water subsystems) mission. The segment parameters for a full transit are as follows; tr_1_: 6 crew, 210 days, tr_2_: 6 crew, 210 days, tr_3_: 2 crew, 500 days, sf: 4 crew, 500 days, asc: 4 crew, 1 day, desc: 4 crew, 1 day. All other configurations are set to their default value. Note that to calculate xESM inventories, technologies that remain on the same craft were scaled to their upper bound of usage. For example, the air processing equipment for the craft throughout tr_1_, tr_2_, and tr_3_ were scaled for 920 days of operation. Consumables (such as stored food) were initially scaled for 920 days and decreased accordingly as they were used.

### Penalty for mission failure

The penalty associated with mission failure can be defined in various ways. For example, we can define the following relationship between the penalty cost and the duration of the mission

19 $$s({u}_{j})=\left(1-\frac{{\hat{t}}_{f}({u}_{j})}{{t}_{tot}}\right),$$

which expresses a linear decrease of the penalization with the number of days.

## Supplementary information

Source Materials

## Data Availability

Source data are provided in this paper.
